# The Multiple Uses of Polypropylene/Polyethylene Terephthalate Microfibrillar Composite Structures to Support Waste Management—Composite Processing and Properties

**DOI:** 10.3390/polym13081296

**Published:** 2021-04-15

**Authors:** Abdulhakim Almajid, Rolf Walter, Tim Kroos, Harri Junaedi, Martin Gurka, Khalil Abdelrazek Khalil

**Affiliations:** 1 Mechanical Engineering Department, College of Engineering, King Saud University, P.O. Box 800, Riyadh 11421, Saudi Arabia; hjunaedi@ksu.edu.sa; 2 Engineering Management Department, College of Engineering, Prince Sultan University, P.O. Box 66833, Riyadh 11586, Saudi Arabia; 3 Institute for Composite Materials (IVW GmbH), Technical University of Kaiserslautern, 67663 Kaiserslautern, Germany; rolf.walter@ivw.uni-kl.de (R.W.); Tim.Krooss@ivw.uni-kl.de (T.K.); martin.gurka@ivw.uni-kl.de (M.G.); 4 Mechanical and Nuclear Engineering Department, College of Engineering, University of Sharjah, Sharjah 27272, United Arab Emirates; kabdelmawgoud@sharjah.ac.ae

**Keywords:** PP/PET microfibrillar composites, waste management, recycling, automotive components

## Abstract

Composite processing and subsequent characterization of microfibrillar composites (MFC) were the focus of this work. Compression molding of wound MFC filaments was used to fabricate MFC composites. The MFC composites were composed of polypropylene (PP) as matrix materials and polyethylene terephthalate (PET) as reinforcement fibers. The PP/PET blends were mixed with PET contents ranging from 22 wt% to 45 wt%. The effect of processing parameters, pressure, temperature, and holding time on the mechanical properties of the MFCs was investigated. Tensile tests were conducted to optimize the processing parameter and weight ratio of PET. Tensile strength and modulus increased with the increase in PET content. PP/45 wt% PET MFC composites properties reached the value of PP/30 wt% GF. Falling weight tests were conducted on MFC composites. The MFC composites showed the ability to absorb the impact energy compared to neat PP and PP/30 wt% GF.

## 1. Introduction

To prevent waste from End of Life Vehicles (ELVs) and to promote the collection, re-use, and recycling of ELVs and their components, the European Union launched the ELV Directive [1]. To achieve the ELV Directive, the automotive industry is working on lightweight material concepts using polymer composites [2]. Most of the polymeric parts used in automobiles for weight reduction are based on fiber-reinforced plastics such as bumpers and dashboards. One of the driving forces for the increased use of fiber-reinforced plastic composites in the automotive industry is weight reduction and recycling ease [3]. Fiber-reinforced plastics are potential candidates to be substituted by microfibrillar composite structures (MFC) [4,5]. Bumpers need to satisfy functional and appearance requirements (they should be able to withstand low-speed impact and have slick appearances). Bumper covers due to traffic accidents are considered the most commonly replaced car body parts [4]. Most polymer bumpers are based on glass fiber-reinforced composites, using polypropylene (PP) or other plastics such as polycarbonate (PC), polycarbonate (PC)/polybutylene perephthalate (PBT), and rubber toughened PP as the matrix [6,7,8,9]. Bumper bars are generally designed to absorb impact energy generated from low-speed impacts (toughened polyolefin (PO) blends or even polyurethane (PU)) [10]. The plastic cover deforms during low-speed impact accidents and pops back into its original shape when heated. The plastic bumper designs have evolved over the years to consider design aesthetics, aerodynamics, cost, and durability issues compared with chromed steel. Polycarbonate and polybutylene terephthalate (PC/PBT) are blended to produce a strong and high-toughness bumper [11,12]. Polycarbonate is a tough thermoplastic, while PBT is an environmental resistance polymer. However, if PC/PBT is struck hard or even hit lightly by a sharp object, it may rupture or tear. Polymer blends are not sufficient for such applications. Instead, more complex structures, e.g., glass fiber-reinforced composites, are used for advanced applications [13,14,15,16]. However, they are, compared with polymer blends, heavier in weight and not easy to recycle [14,17].

Recent studies on the mechanical properties of compression-molded (CM) and unidirectionally arranged MFC composites found that the flexural modulus and strength are higher than those of injection-molded MFC composites [18,19,20,21]. The values of the mechanical properties of compression-molded (CM) are even higher than those of short glass fiber-reinforced PP [22,23]. These experimental results demonstrate the strong effect of the length, the aspect ratio, and the alignment (orientation) of the reinforcing fibrils on the mechanical properties of the MFCs.

For the polyethylene terephthalate (PET)/ low density polyethylene (LDPE) system, the composites’ tensile properties were found to be comparable to the tensile properties of short glass fiber-reinforced LDPE [24,25,26]. The improvement in tensile properties is related partially to the formation of trans-crystalline layers of LDPE on the PET microfibril surface, which is observed for the first time on lamellae level [27,28,29]. In PET/PP, our earlier studies showed that the tensile properties (strength, modulus) were also outstanding compared to the respective neat materials [23,30].

In our previous research work [30], we presented and adapted the concept of microfibrillar composites (MFC). The purpose was to produce composites of polyethylene terephthalate (PET) fibers reinforced polypropylene (PP) materials. The relationship between the morphology of the MFC structure and the mechanical behavior of the MFC filament was investigated. Meanwhile, in this research and as a second step, our primary goals of this investigation are: (i) to apply the experience gained on the MFC procedure for manufacturing of different polycondensate/polyolefine blends based on PET/PP and to achieve improved mechanical and significantly impact properties, appropriate for the production of car bumpers; and (ii) to transfer the gained know-how into an industrial production process. In the blends, PET fibrils play the reinforcing components’ role while PP forms the matrix phase. The relationship between the mechanical properties of test specimens with an MFC structure was characterized. The behavior analysis helps to understand the influence of, e.g., the mold temperature, mold pressure, and holding on the PP/PET MFC composites’ properties. The impact properties of the PP/PET MFC composites are also investigated. Finally, it should be underlined that all proposed blends are entirely recyclable and can be re-used. The novelty of the recent work is to prevent waste at the end-of-Life Vehicle (ELV) and to promote the collection, re-use, and recycling of ELVs and their components.

## 2. Materials and Methods

### 2.1. Materials

The microfibrillar composite was prepared from PET filler in a PP matrix. The raw materials were manufactured by SABIC Company (Riyadh, Saudi Arabia). The physical properties of the MFC constituents are presented in Table 1.

### 2.2. Blending and Filament Processing

The processing of the MFC filaments started with dry mixing of PP/PET with different PET contents ratios ranging from 22–45 wt%. The polymer mix was extruded using a twin-screw extruder (PL2000, Brabender GmbH&Co., KG Duisburg, Germany). The extruded filaments were immersed in a water bath to cool the filaments to room temperature with a speed-controlled drive unit with initial speed *v*_1_. The extruded filament was led into the stretching chamber, where the temperature was kept above the *Tg* of all used polymers. The filament was continuously stretched. The stretching ratio was controlled by a second drive unit with a speed *v*_2_. Finally, the stretched filaments were wound up on a spool. The detailed processing of the MFC filament was discussed in our earlier work [30].

### 2.3. Composite Processing

The isotropization of MFC filament in which the PP fibrils transformed at high temperature into an isotropic polymer matrix reinforced with fibril of PET is realized during the production of composite plates through Compression Molding Process (CM). A laboratory-scale two-column Paul Weber hot press (PW10H-HKP300-ø165, Remshalden, Germany) was used to develop the MFC composite plates.

**Figure 1:**
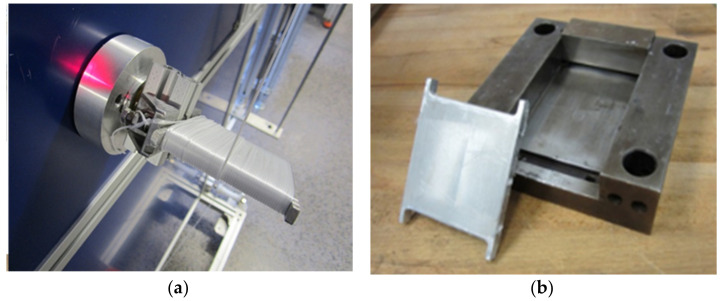
Production of MFC composite plate, (**a**) filament winding around the aluminum plate, (**b**) compression hot press mold.

The composite plates were manufactured with the following procedure:

1. Winding filaments around an aluminum frame using the filament spooling unit (Figure 1a).
2. Inserting the frame with several layers of filaments into a hot press mold (Figure 1b).
3. Hot-pressing cycle in pressure and temperature controlled hot press.

Once the hot press mold had been cooled to room temperature under pressure, the frame with the MFC plates on each side was removed from the press, and plates of constant thickness were cut out. Thus, a plate that was oriented unidirectionally with the dimensions 80 × 80 × (1–2.5) mm^3^ and with an isotropized matrix was developed.

**Figure 2:**
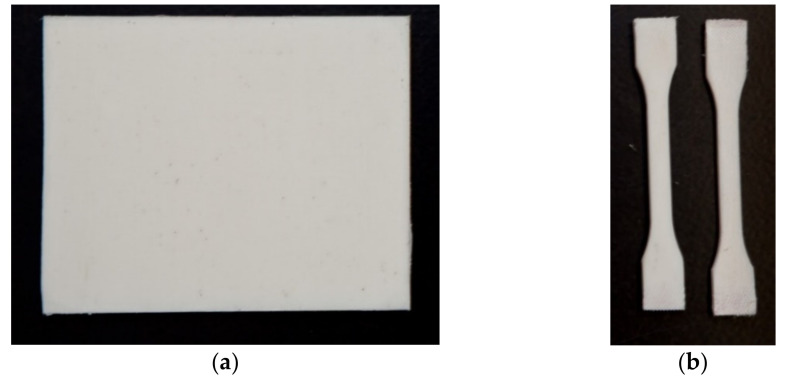
Testing samples (**a**) MFC plate, and (**b**) “dog-bone” standard samples.

Testing samples were prepared by cutting the testing block using a diamond saw to the desired testing shapes. Figure 2 shows the MFC plate for impact testing and dog-bone specimens for tensile testing.

### 2.4. Mechanical Characterization

#### 2.4.1. Tensile Test

The MFC compression-molded specimens were tested in a universal testing machine (Zwick 1474, Ulm, Germany). The tensile tests were conducted according to the DIN EN ISO 527 standard. The tests were conducted at room temperature with a crosshead speed of 5 mm/min. The modulus of elasticity was measured using an extensometer placed on the testing sample during the elastic region.

#### 2.4.2. Impact/Falling Weight Test

**Figure 3:**
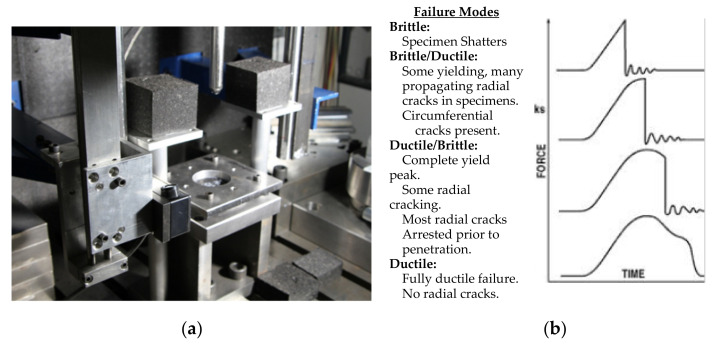
(**a**) Falling Weight Test Device, (**b**) modes of failure.

The Instrumented Falling Weight Impact (IFWI) tester (Model Fractovis 6785, Ceast, Torino, Italy) shown in Figure 3a was used to measure the response assessment of out-of-plane fracture for polymers and composites. Figure 3b shows possible failure responses for different falling weight impacts. IFWI provides a realistic prospect of the material response against impact loads that fall perpendicular to the filament direction. IFWI tests were conducted according to the EN ISO 6603 standard. Tests were realized at a speed of 4 m/s, spherical indenter of 20 mm diameter, plate size of 80 mm × 80 mm. The plate was mechanically clamped at room temperature. Force-displacement (*F* vs. *x*) curves were recorded, while energy-displacement (*E* vs. *x*) curves were calculated and reported.

The maximum force in the force-deflection curve is related to the crack initiation. The integration of the force over the displacement up to the maximum force (*x* at *F_max_*) resulted in the normalized energy at crack initiation [31].

(1) $$E_{ini}=\frac{1}{t}\int_{0}^{x_{max}}F\;dx,$$

where *t* is the sample thickness, *F* is the impact force, and *x_max_* is the maximum force-displacement. Similarly, the integration of force over the displacement for the total displacement provides the normalized total energy required to fully penetrate the specimen,

(2) $$E_{tot}=\frac{1}{t}\int_{0}^{x_{total}}F\;dx,$$

where *x_total_* is the total displacement. Ductility index (*DI*) is a parameter that measures the impact perforation of the material and is measured as:

(3) $$DI=\frac{E_{tot}-E_{ini}}{E_{tot}}=\frac{E_{prop}}{E_{tot}},$$

where *E_prop_* is the energy required for disk penetration.

## 3. Results and Discussion

**Figure 4:**
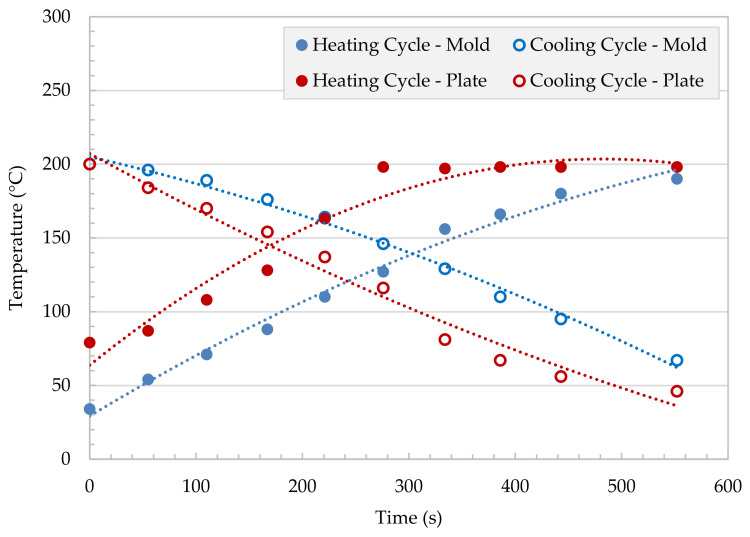
Plate processing by hot press cycle: Accuracy of mold heating/cooling PP/PET.

Preliminary investigations on the lag time between the applied temperature and the actual temperatures inside the mold and the press plate were conducted. Heating and cooling cycles were performed, and they showed a constant delay between the mold and the press plate temperature of about 90–200 s. Figure 4 shows the lag time between the press plate temperature and mold temperature as a function of time. The lag time decreases with increasing temperature. The typical pressing times are in the range 1000–6000 s. For this reason, this delay is minimal and can be neglected.

The MFC filaments are highly oriented and have high strength and modulus due to the matrix’s anisotropy [32,33]. Isotropization transforms the matrix and turns the PP into isotropic structure. As the temperature increases, the degree of isotropization increases, which results in lower strength and modulus [25]. The concept of isotropization will be used in developing composite materials. Isotropization will be realized during compression molding.

### 3.1. Tensile Test

**Figure 5:**
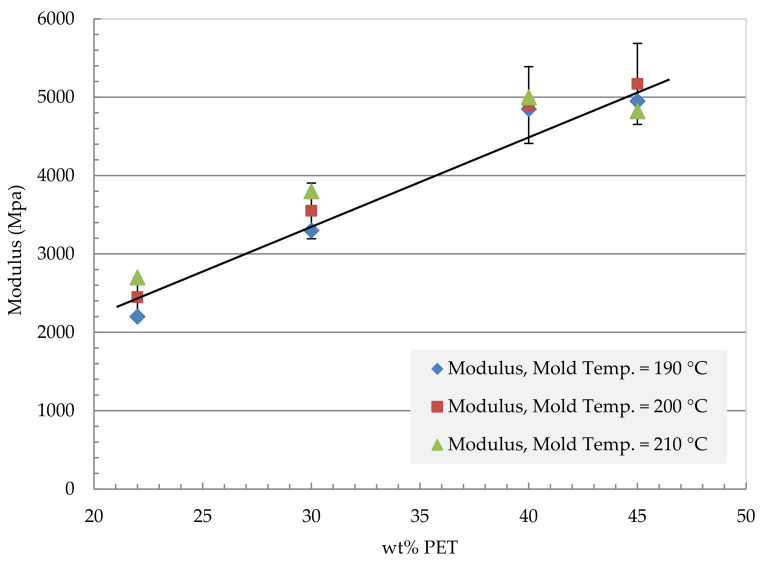
Mechanical properties of MFC tensile modulus vs. wt% PET, applied pressure 10 MPa, holding time 4200 s.

**Figure 6:**
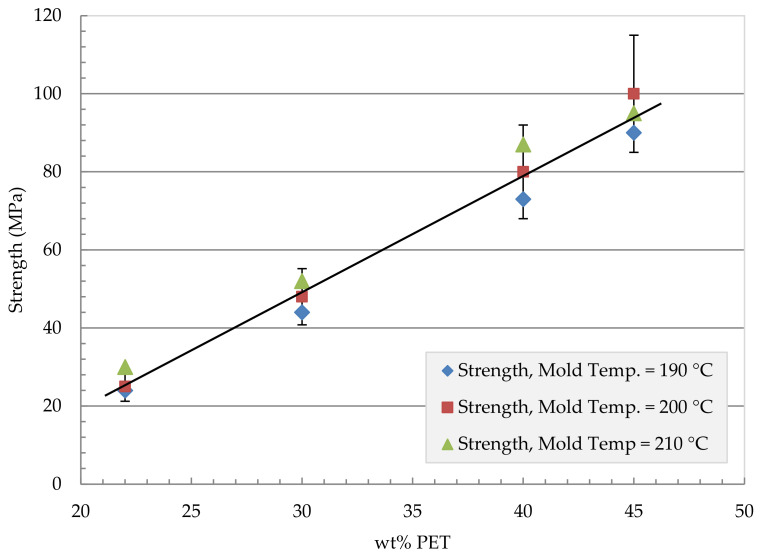
Mechanical properties of pressed plates: Strength vs. wt% PET, applied pressure 10 MPa, holding time 4200 s.

**Figure 7:**
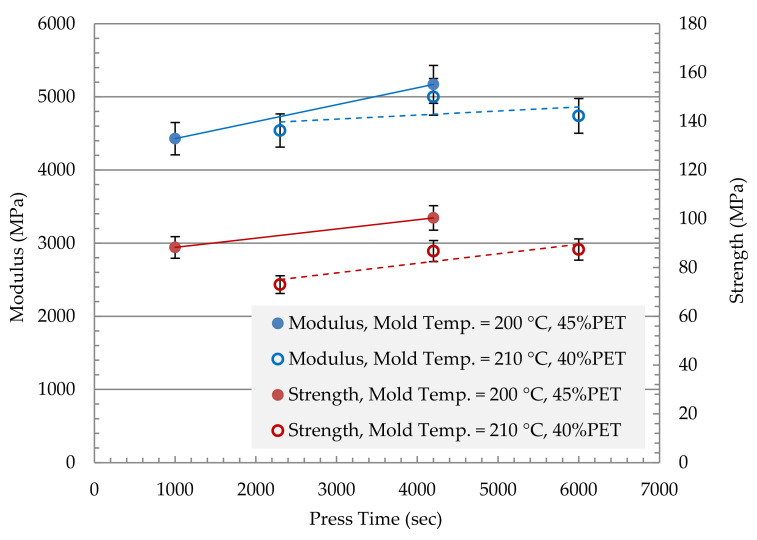
Mechanical Properties of PP + 40, 45% PET: Modulus + Strength vs. Pressing time, applied pressure 10 MPa.

**Figure 8:**
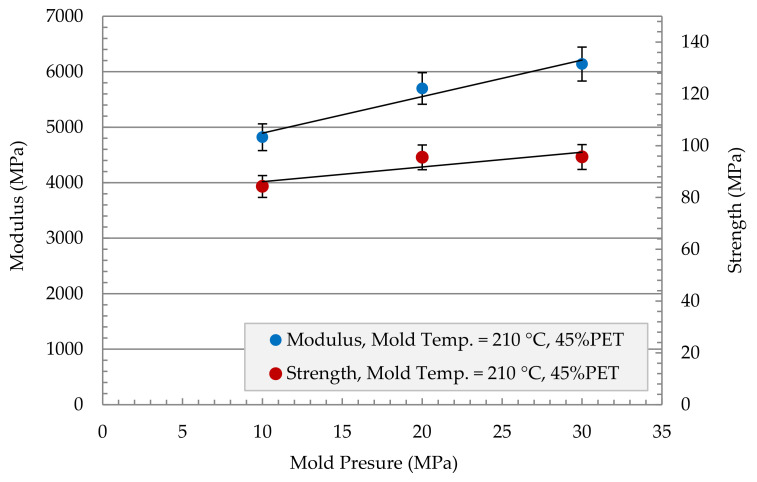
Mechanical properties of PP + 45% PET Modulus + Strength versus Mold Pressure, holding time 1000 s.

The mechanical properties (tensile strength and modulus) of the compression-molded unidirectional fibrillar reinforced plates with different compositions and manufacturing conditions are presented in Figure 5, Figure 6, Figure 7 and Figure 8. The compression molding parameters are temperature, pressure, and holding time. Figure 5 shows the effect of the mold temperature on the MFC tensile modulus at different PET volume fractions ranging from 22 wt% to 45 wt% PET. The mold pressure was held at 10 MPa, and the holding time was 4200 s. The modulus has increased with the increase in PET content. The modulus increased from 2400 MPa at 22 wt% PET to about 5200 MPa at 45 wt% PET. Similarly, Figure 6 shows the effect of mold temperature on the MFC tensile strength. The plate was held to the same mold pressure and holding time at 10 MPa and 4200 s, respectively. A mold temperature of 200 °C gives the highest strength in the MFC plate at 45 wt% PET.

MFC plate at 40% and 45% PET showed the most significant improvement in terms of strength and modulus. Further investigations on the effect of press time and applied pressure are conducted.

Figure 7 shows the effect of press time on the properties of the MFC plate. The two compositions that showed promising results (40% and 45% of PET) were further investigated to the effect of press time. The mold temperature for the 40 wt% PET plate was selected as 210 °C as it has shown to give the best result (Figure 5 and Figure 6). At the same time, the mold temperature for the 45 wt% PET was selected as 200 °C. The applied pressure was maintained at 10 MPa, and the test was conducted at two different mold temperatures: 200 and 210 °C. As the press time increases, both modulus and strength increase. The 45 wt% PET showed the best result at a mold temperature of 200 °C. The maximum stress of 100 MPa was obtained for 45 wt% PET at a mold temperature of 200 °C and press time of 4200 s, and pressure of 10 MPa. The maximum modulus of 5200 MPa was obtained at the same parameters as the maximum stress case for 45 wt% PET at a mold temperature of 200 °C and press time of 4200 s, and pressure of 10 MPa.

The effect of mold pressure on modulus and strength was investigated for the MFC plate of 45 wt% PET in Figure 8. The mold was heated to 210 °C, and a 1000 s holding time was applied. Both modulus and strength experienced an increase in their value as the mold pressure increase. The modulus increased from 4800 MPa at 10 MPa mold pressure to 6200 MPa at 30 MPa mold pressure, which corresponds to a 30% increase in modulus. The strength did not show that sharp increase as it increased from 85 MPa to 95 MPa in the two mold pressure ranges, which corresponds to a 12% increase in strength.

**Figure 9:**
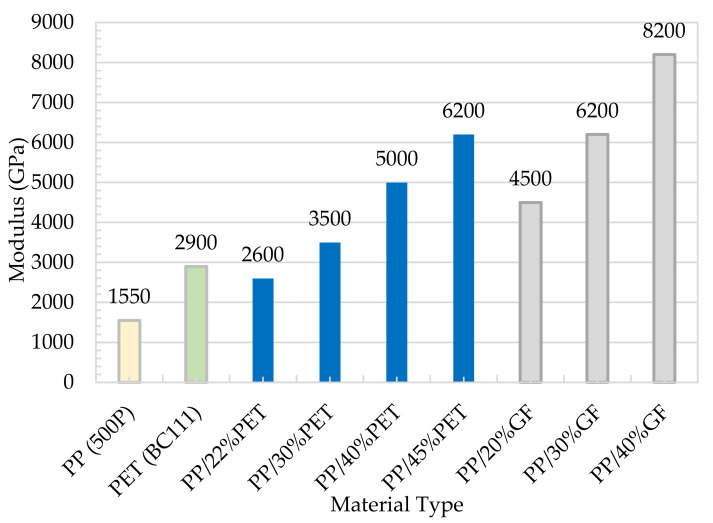
Modulus of the neat polymer, MFC composites, and PP/GF.

**Figure 10:**
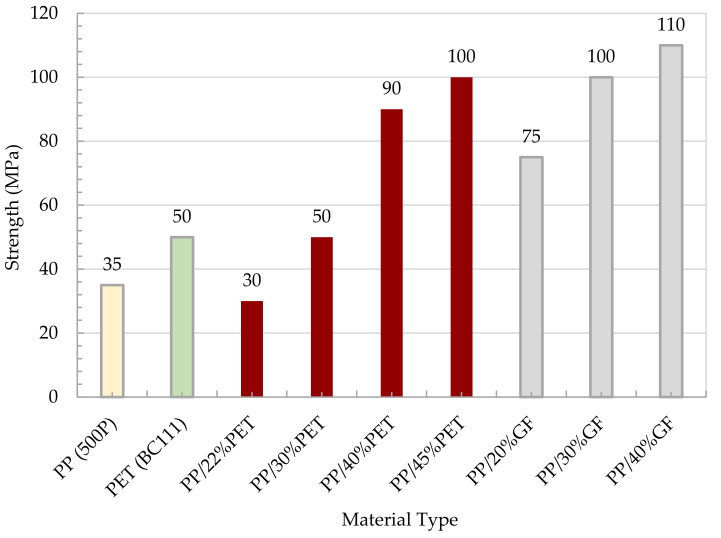
Tensile strength of the neat polymer, MFC composites, and PP/GF.

Figure 9 shows a comparison in terms of modulus between the MFC plate with different compositions and the raw polymers of PP and PET. The figure also shows a comparison with PP/Glass Fiber composites. The modulus of MFC at 45 wt% PET reached 6200 MPa which is higher than the neat PP and PET. The MFC modulus at 45 wt% PET is higher than PP/20 wt% GF and is similar to PP/30 wt% GF. A similar comparison is presented in Figure 10 for tensile strength. The strength of 45 wt% PET reached 100 MPa, which is close to PP/30 wt% GF. PP/PET microfibrillar composites are great candidates to replace PP/GF in many applications. The strength and modulus data used in Figure 9 and Figure 10 are the average data obtained in Figure 5, Figure 6, Figure 7 and Figure 8. The use of MFC will improve the waste management and sustainability of industrial components and products, which contributed to the End of Life directive of the European Union.

### 3.2. Impact/Falling Weight Test

#### 3.2.1. Sample Preparation

**Figure 11:**
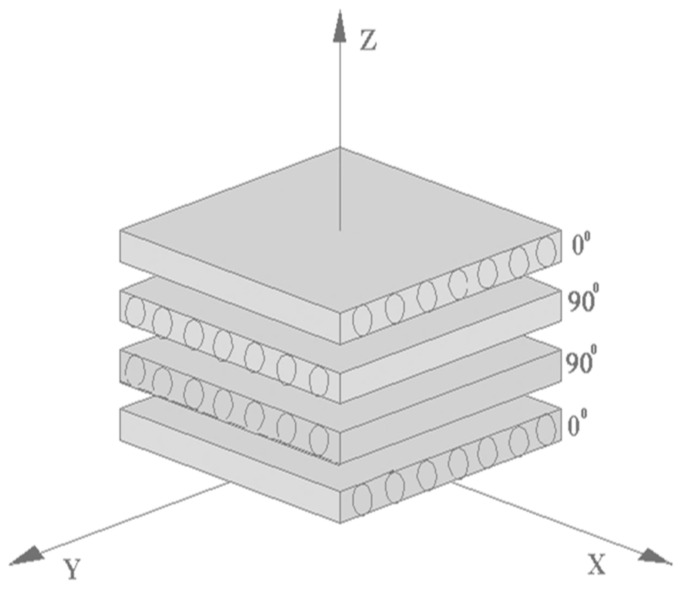
The schematical orientation of microfibrillar reinforced composite plates.

The samples for falling weight tests were prepared from four plates of unidirectional fibrillar reinforced PP plates of 1 mm thickness in a hot-press mold. For the preparation of a quasi-isotropic plate, the individual plates were arranged according to the layup design of fiber-reinforced composites with 0°/90°/90°/0° layup shown in Figure 11. The determination of specific fracture energies considered the differences in the total plate thicknesses of the manufactured plates. The goal was to determine the potential use of the fibrillar reinforcement to improve MFC’s impact behavior.

The asymmetric composite layup was selected to minimize warpage in the MFC structures and to have bidirectional strength in the MFC system. The plates were pressed with the optimized parameters determined for unidirectional plates at a temperature of 200 °C, pressure of 10 MPa, and a press time of 4200 s.

#### 3.2.2. Impact/Falling Weight Test

**Figure 12:**
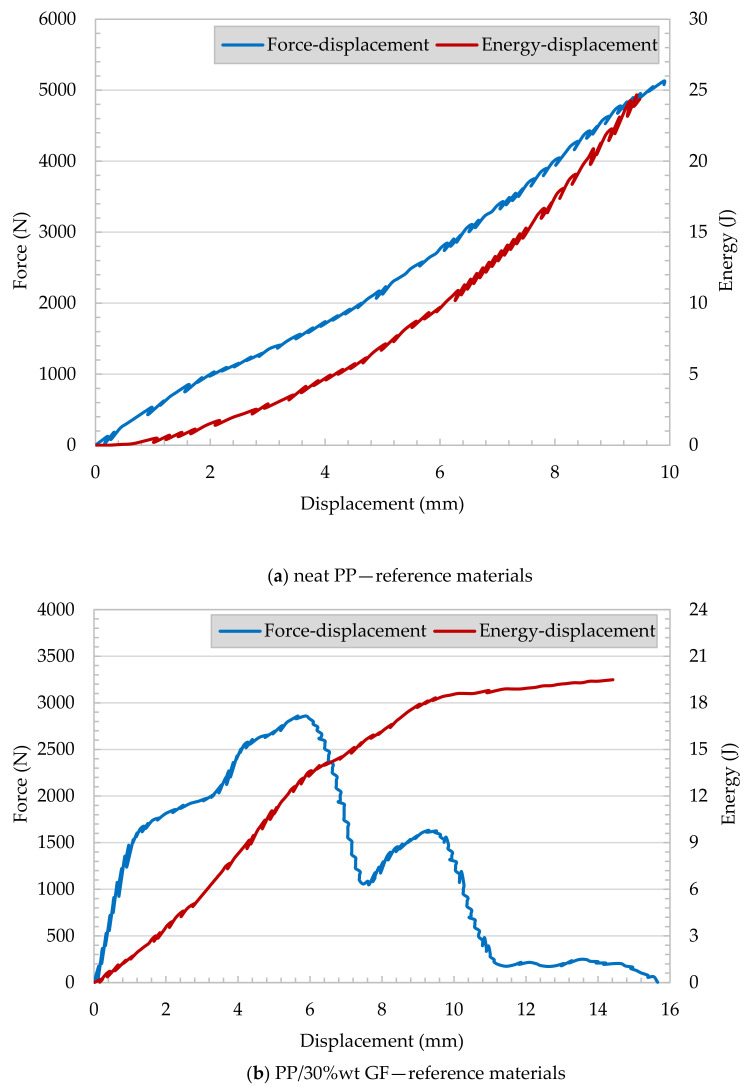
Impact behavior of reference materials ((**a**) neat PP and (**b**) PP/30 wt% GF).

Figure 12 shows records from the tested injection-molded reference plate. Injection-molded neat PP and PP/30 wt% GF samples underwent falling weight impact tests. The force versus displacement and energy versus displacement curves of neat PP represents a brittle failure, as shown in Figure 12a. No yield or plastic deformation can be observed. In Figure 12b, the energy versus displacement of PP/30 wt% GF shows a continuous increase in the calculated energy with respect to the reasonable increase of displacement. Meanwhile, the force increased up to certain limit (mainly at 2.7 kN) and then dramatically decreased. This can be attributed to crack initiation with further energy consumption associated with the crack propagation.

**Figure 13:**
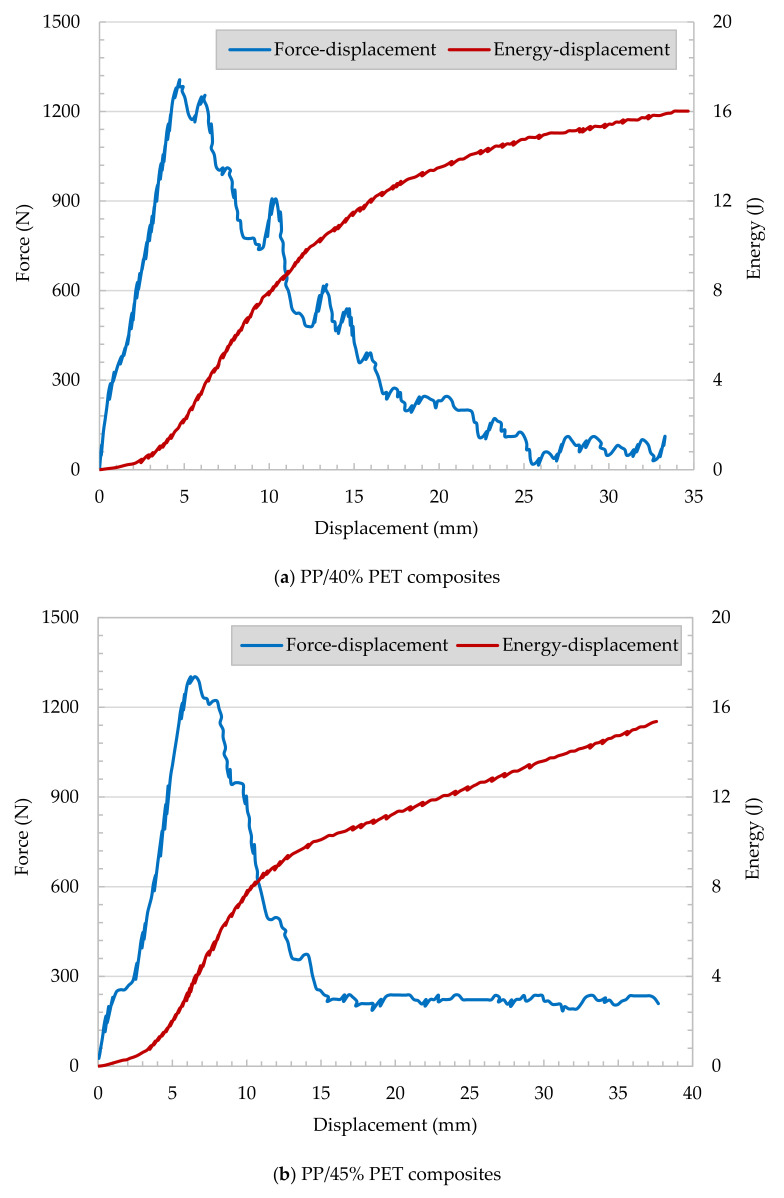
Impact behavior of MFC plates ((**a**) PP/40 wt% PET and (**b**) PP/45 wt% PET).

The records of PP/PET fibrillar reinforced plates (Figure 13a,b) show similar performance compared to PP/30 wt% GF composites. The samples appear to have energy consumption during crack propagation.

The penetration force of neat PP shows a linear increase till the maximum force of 1.3 kN is reached at a displacement of 5 mm, followed by dramatic decrease due to crack propagation. It can be concluded that glass fiber- and fibrillar-reinforced plates show pronounced crack propagation and high energy consumption. Characteristic impact parameters are summarized in Table 2.

**Figure 14:**
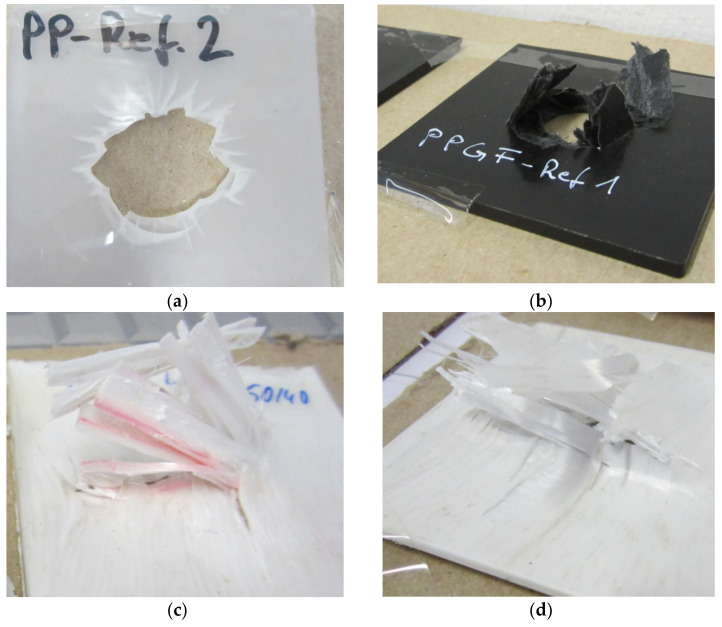
Fractured impact samples, (**a**) neat PP, (**b**) PP/30 wt% GF, (**c**) PP/40 wt% PET MFC, and (**d**) PP/45 wt% PET MFC.

The specific crack initiation energy appears to be very high for the neat PP, and a medium level was determined for PP/30 wt% GF. The crack propagation range’s energy absorption values are higher for MFC and GF reinforced plates compared to the neat PP. It appears that for the MFC plates, the specific crack propagation energy is in the same range as for PP/30 wt% GF, and due to brittle failure, the PP consumes no propagation energy. As shown, the ductility index (*DI*) is the fraction of the crack propagation energy of the total work required to fracture the specimen. Neat PP offers the lowest ductility index of all tested materials. The MFC reinforcements have a higher ductility index compared to PP/30 wt% GF. Detailed investigations of the impacted sample (Figure 14) can clarify the various failure mechanisms. The impact fracture of neat PP is brittle, and the specimen was shattered fracture due to the high rigidity and crystallinity of the PP, as shown in Figure 14a. PP/30 wt% GF showed some brittle/ductile fracture performance (Figure 14b). There is a slight yielding occurred, but still, some sample shattering exist. In MFC plates (PP/40 wt% PET and PP/45 wt% PET, Figure 14c,d), some ductile/brittle fracture with yielding occurred. The material is intact, and no shattering occurred.

## 4. Conclusions

Manufacturing of different polycondensate/polyolefine blends based on PET/PP was conducted to achieve improved mechanical and especially impact properties, appropriate for the production of car bumpers. In the blends, PET fibrils were used in the role of reinforcing components, while PP formed the matrix phase. The relationship between the mechanical properties of test specimens with an MFC structure was characterized. Considerably improved tensile properties (300% increased) could be achieved with PET fibril contents of 40% and 45 wt%. Reliable isotropization of these materials without reduction of fibrillar reinforcement can be achieved with mold temperatures 200 °C and 210 °C. Increased properties were measured for higher mold pressure (30 MPa). The mechanical characterization led to optimized press parameters for 40 wt%, and 45 wt% PET-filled plates: mold temperature 200 °C, applied pressure 10 MPa, holding time 4200 s. The influence of the applied pressure for a reduced holding time of 1000 s shows the best mechanical properties for PP + 45 wt% PET MFC plates: Mold temperature 210 °C, mold pressure 30 MPa. The improvement in the MFC mechanical performance reached that of PP/30% GF. The MFC composites showed great falling weight performance. The MFC has high crack propagation energy similar to PP/30 wt% GF. Finally, it can be concluded that all proposed blends are entirely recyclable and can be re-used.

## Figures and Tables

**Table 1:** Manufacturer’s datasheet of MFC-based polymers.

Polymer Type	Polymer Grade	Manufacturer	Density (g/cm^3^)		MFR (dg/min)	Intrinsic Viscosity (dL/g)
			Bulk	Crystalline		
PET	BC111	Sabic	0.838	1.39	-	0.74–0.78
PP	500P	Sabic	-	0.905	3	-

**Table 2:** Characteristic parameters of impacted PP, PP/PET composites, and PP/30 wt% GF.

% PET	Densityg/cm^3^	Thickness(mm)	*F_max_* (N)	Standard Deviation, *F_max_*	Displ. at *F_max_* (mm)	Standard Deviation, *Disp.*	Energy at *F_max_* (J/mm)	*E_tot_* (J/mm)	Specific *E_tot_* (J/g)	Ductility Index (*DI*)
0	0.905	3.9	5440	±200	11.3	±1.3	8.4	8.52	0.23	0.02
40	1.1	4.07	1449	±50	7.0	±0.5	2.42	6.59	0.147	0.61
45	1.12	3.08	1309	±40	6.3	±0.4	2.49	7.76	0.223	0.67
30 wt% GF	1.15	4.0	3388	±120	5.2	±0.27	4.58	9.07	0.197	0.48

## Data Availability

The data presented in this study are available on request from the corresponding author.
